# A Rare Case of Posterior Vaginal Wall Gartner's Duct Cyst Mimicking as Genital Prolapse

**DOI:** 10.7759/cureus.31507

**Published:** 2022-11-14

**Authors:** Sharmeen I Memon, Neema Acharya

**Affiliations:** 1 Department of Obstetrics and Gynaecology, Jawaharlal Nehru Medical College, Datta Meghe Institute of Medical Sciences, Wardha, IND

**Keywords:** inclusion cyst, bartholin's cyst, uterovaginal prolapse, posterior vaginal wall cyst, gartner's duct cyst

## Abstract

A cyst is a blind pouch of tissue, which may be containing air, fluid, pus or any other substance. Vaginal wall cysts are located on or under the vaginal wall lining, are usually asymptomatic and may present as a small lump felt in the vagina or protruding from the vagina. These cysts can be of varying sizes, ranging from the size of a pea to that of an orange. These may get infected and present with inflammatory signs and symptoms.

We report here a case of a 38-year-old female who presented with complaints of something coming out of her vagina, initially mistaken to be uterovaginal prolapse, which, on detailed clinical examination, was diagnosed to be a posterior vaginal wall cyst. The cyst was managed surgically by excision, and on histopathological examination, it was later confirmed to be a Gartner's duct cyst over the posterior vaginal wall, rare in its location.

## Introduction

Cysts of vaginal origin are rare. Several categories of cysts have been identified and are classified based on histology, that is, according to the lining epithelium of the cyst wall: epidermal inclusion cyst, Mullerian cyst, endometroid cyst, and Bartholin cyst or abscess; Gartner’s duct cyst; and unclassified types [1]. Gartner's duct cysts are found in about 20% to 25% of women, out of which nearly 1% of women develop Gartner's duct cysts. They arise as a consequence of an obstruction of Gartner’s duct (remnant of the mesonephric duct) and commonly arise from the anterior vaginal wall. Posterior vaginal wall Gartner's duct cysts are very rarely found, with an incidence rate of about 12.5% of all vaginal wall cysts [2].

## Case presentation

A 38-year-old female (obstetric score: parity 2, live issues 2), with a history of previous two full-term vaginal deliveries, presented with a complaint of a mass protruding from the vagina for the last one year. Initially, the mass was very small, the size of a peanut, which progressed gradually to the size of a lemon, resulting in discomfort to the patient in doing day-to-day activities. The mass was not reducible on lying down and did not increase in size on straining or coughing.

She had a history of on-and-off vague abdominal discomfort for the last six months. The history of past menstrual cycles was normal, with 3 to 4 days of menstrual bleeding every 28 to 30 days, regular with the average flow and no complaints of the passage of clots or dysmenorrhoea.

**Figure 1:**
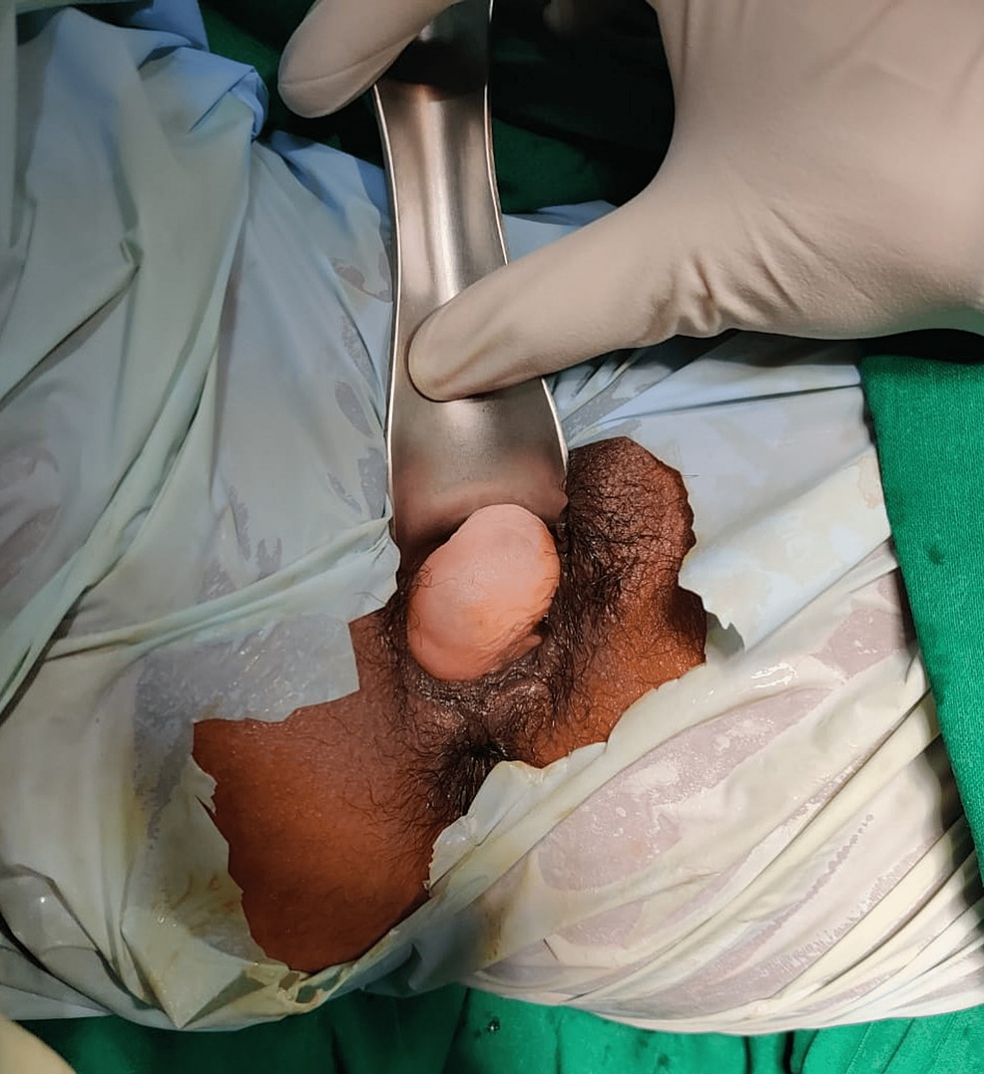
Per speculum examination showing the posterior vaginal wall Gartner's duct cyst.

The patient was vitally stable with normal general and systemic examination findings. On per speculum examination, a posterior vaginal wall cyst of approximately 3 cm × 3 cm in size and pale pink with a smooth intact surface was protruding out of the vaginal introitus. The cyst was non-tender, mobile and fluctuant (Figure 1). The anterior and lateral vaginal walls were unremarkable. The cervix was seen high up on speculum examination distant from the inner border of the cyst. On per vaginal examination, it was found that the uterus was normal in size, anteverted, anteflexed and mobile. Bilateral fornices were free. On straining, no cystocele or rectocele was demonstrated. Rectal mucosa was free with a smooth and normal anterior rectal wall, on per-rectal examination.

Her investigations show haemoglobin 10.6 g/dL, total leukocyte count 7,600 mm^-3^, platelet count 3.40 lacs/mm^3^, Na^+^ 137 mmol/L, K^+^ 4.3 mmol/L, serum creatinine 0.53 mg/dL and cancer antigen 125 (CA 125) 12 units/mL (normal range 0-35 units/mL).

**Figure 2:**
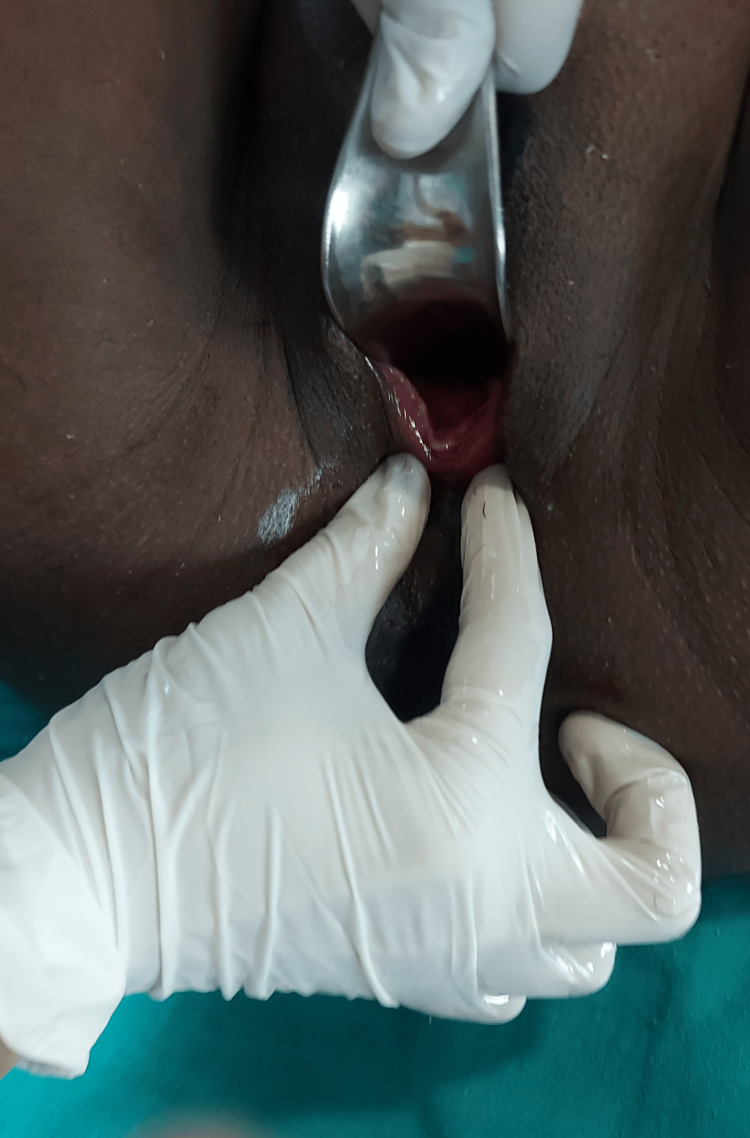
Post-operative examination after excision of the cyst.

Excision of the cyst was planned under all aseptic precautions using intravenous sedation with lignocaine 2% + adrenaline injected around the base of the cyst. The posterior vaginal wall was dissected from the fourchette upwards. The base of the cyst was identified. The incision is taken over the most dependent part of the cyst. Cystic fluid was drained. Planes were separated, and the cyst wall was excised from the posterior vaginal wall and sent for a histopathological examination. Per-rectal examination was done to ensure the integrity of the posterior vaginal wall and anterior wall of the rectum. The posterior vaginal wall was sutured with Vicryl 2-0 (Polyglactin, Aspiron, Meril Endo Surgery Pvt. Ltd., Mumbai, India), and hemostasis was ensured. Vaginal packing was carried out. The patient withstood the procedure well. The post-operative period was uneventful (Figure 2).

**Figure 3:**
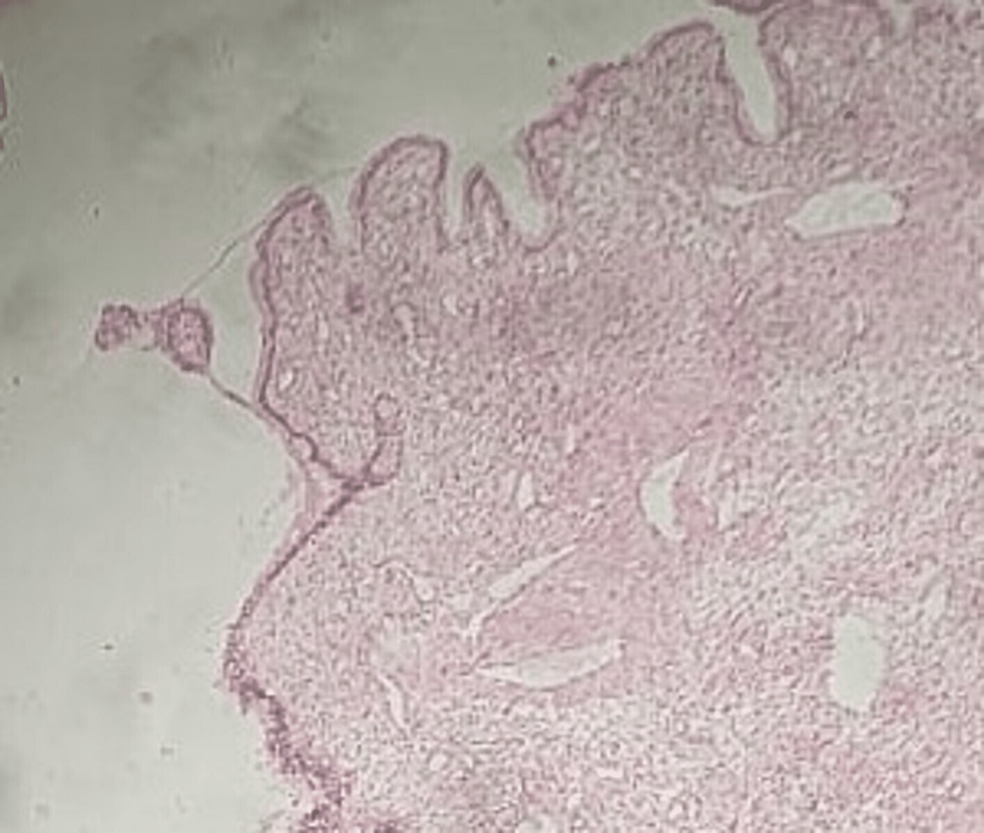
H- and E-stained photomicrograph showing the cyst wall lined by cuboidal to columnar epithelium.

The histopathology report showed tall cuboidal to columnar epithelial lining of the cyst wall (Figure 3).

## Discussion

Gartner's ducts are found in approximately 25% of women, of which nearly 1% transform into the formation of Gartner's duct cysts [3]. These cysts usually do not present with symptoms and are an incidental finding on routine gynaecological examination [4]. But a few patients may present with complaints of dyspareunia, dysuria, voiding disturbances, vaginal discharge, itching, pelvic pain and sometimes with visible palpable mass [5,6]. During the period of embryological development, there occurs a regression of the Wolffian ducts in females, but in rare cases, a secretory mechanism develops in the remnants, which causes dilatation of the surrounding cells, resulting in the formation of a Gartner’s duct cyst [7]. The usual location of Gartner's duct cysts is on the anterior or lateral vaginal wall. These are usually solitary, unilateral and less than 2 cm in diameter and may very rarely present over the posterior vaginal wall, as seen in this case [8].

Various differential diagnoses, as enumerated in Table 1, were considered during the evaluation and were ruled out by the clinical examination [9]. To differentiate Gartner’s duct cysts from other pathologic vaginal cysts and swellings, magnetic resonance imaging can be a modality of investigation [10,11]. The histopathological examination helps to exclude other differential diagnoses by correctly identifying the cyst wall lined by low columnar or cuboidal epithelium [12,13]. Malignant transformation is seen in exceptionally rare cases.

**Table 1:** List of differential diagnoses of vaginal wall cysts.

Differential diagnosis of vaginal cysts
Bartholin gland cyst or abscess
Inclusion cyst
Endometriotic cyst
Mesonephric cyst
Cystocele
Rectocele
Enterocele
Haematocolpos
Myxomatous cyst

## Conclusions

Large posterior vaginal wall Gartner's duct cysts are symptomatic and cause discomfort to patients, compelling them to visit gynaecologists, which can be easily mistaken to be a case of uterovaginal prolapse. Clinical examination solves the confusion. Treatment includes conservative management or surgical excision. The histopathological examination helps confirm the diagnosis. Thus, not all patients who present with mass coming out of the vagina have to be a case of uterovaginal prolapse.
